# Immune and non-immune congenital heart block: a tale of two very different entities

**DOI:** 10.1093/europace/euaf041

**Published:** 2025-03-01

**Authors:** Susan P Etheridge, Janette F Strasburger

**Affiliations:** Division of Cardiology, Department of Pediatrics, Children’s St. Lukes Hospital, Boise, ID 83712, USA; Stanford Lucile Packard Children’s, Palo Alto, CA 94304, USA; Professor of Pediatrics and Biomedical Engineering, Medical College of Wisconsin and Children’s Wisconsin, Milwaukee, WI, USA

**This editorial refers to ‘Late outcomes of congenital and childhood non-immune, isolated atrioventricular block: a French nationwide retrospective cohort study’, by F. Mycinski *et al*., https://doi.org/10.1093/europace/euaf040.**

Congenital atrioventricular (AV) block although rare, represents an important part of what we do as foetal cardiologists and paediatric electrophysiologists. These are our smallest, youngest, and often our most challenging patients. Significant technical advances in the care of this population have been made in the last decade. Pacing is undertaken in increasingly smaller infants and with more physiologic pacing strategies. Modifying a leadless pacing device made for an adult to be an epicardial device has extended pacing reach to tiny premature infants. Identifying optimal epicardial and endocardial lead locations helps prevent pacing-induced cardiomyopathy in a child who can require > 50 years of pacing.^1,2^ While our understanding of immune-mediated congenital AV block has matured, the rarer patient with non-immune congenital AV block is less well studied. Are the outcomes of this more diverse non-immune population the same as in the immune congenital AV block population? Mycinski *et al*. in this issue of Europace^3^ expanding on their 2012^4^ data, help untangle the aetiologies and modern-day outcomes in children with non-immune congenital AV block. Although over 90% of very young onset AV block is immune-mediated, Mycinski *et al*. present data on the much rarer non-immune population.^3^ This is an impressive collaboration from 29 tertiary children’s hospitals, likely encompassing nearly all such entities in France. They detail 42 years of patient data in 385 non-immune AV block cases, gathered to advance our knowledge of this rare entity. The category non-immune and isolated congenital AV block includes only those children without a maternal isoimmune or a patient-identified aetiology of the AV block. Is this distinction important? Yes, as the authors of this article prove, outcomes are not the same, management is different, aetiologic assessment imperative, and knowledge is power.

Immune-mediated heart block can result in long-term sequelae, encompassing immune-mediated myocardial injury or transient myocarditis and cardiomyopathies with dilated cardiomyopathy seen in in 15% to 30%.^5,6^ In the paper by Mycinski *et al*.,^3^ we find a reassuring outcome for those with non-immune AV block where after a median follow-up of 10 years, no patient died, developed endomyocardial fibrosis, or dilated cardiomyopathy. Most received pacemakers and yet there were no patients recognized as having pacemaker-mediated cardiomyopathy. This is consistent with data from Sagar *et al*. who demonstrated that natural history of patients with isolated congenital AV block who require pacing depended upon their antibody status.^7^ Antibody status was a predictor for the development of heart failure and death. Long-term right ventricular pacing alone does not appear to be associated with development of heart failure, deterioration in ventricular function, or reduced survival in antibody negative isolated congenital AV block patients. The authors conclude that antibody status has a more significant impact on the development of heart failure than prolonged right ventricular pacing, with a potential immune-mediated process leading to heart failure. Thus, establishing an aetiology for heart block in children is imperative if we are going to wisely manage patients and counsel families concerning long-term risk.

In this article, we are reminded that this is a disease of the very young with a median age at diagnosis of 3 years and a subset of 34 infants who presented *in utero* or at birth. This article furthers our understanding of the contribution of genetics (recognized and unrecognized) to young onset AV block. In this large study, 22 (5.7%) of subjects presented prenatally, an additional 12 (3.1%) at birth or in the first month of life. While presumably, not all of these had inherited conduction system disease some proved to have a genetic variant that could be responsible. Some family members were assessed, and their data corroborate a genetic basis for the AV block. Although few family members had symptoms or identified disease, of the 204 parents who underwent an ECG screening, conduction abnormalities were found in 78 (38.2%), including first degree and unrecognized complete AV block, bundle branch block and QT prolongation. Due to the eras encompassed by the data in this paper, we can be certain that not all patients were tested, testing was less sophisticated, gene discovery less mature, and gene coverage less broad. Likely, if performed today, a genetic aetiology for the AV block would be identified far more frequently. This is further enforced by the progressive nature of the AV block in this series where paroxysmal block progressed to permanent and incomplete to complete AV block. Familial progressive cardiac conduction system disease is in the early stages of gene discovery. Recent developments in molecular biology and genetic technologies have enabled the discovery of genetic basis of some forms and current knowledge suggests that it often results from variants in genes encoding cardiac ion channels, involved in cardiac electrical impulse propagation.^8^ Even today a substantial proportion of these patients test negative for alterations in currently discovered progressive cardiac conduction-related genes, suggesting that many genes causally involved are yet to be discovered.

The concept that a potentially lethal genetic and inherited disorder can manifest in early life underscores the importance of its recognition, management, and the timely initiation of cascade family testing. We now know that the foetus can be the proband and if identified can result in life-saving patient identification and pre-symptomatic therapy in the family.^9^ This is supported by the findings of the large Japanese study of infant Long QT Syndrome (LQTS), where 62% of cases with LQTS presented *in utero* or within 2 days of life, many with second degree AV block.^10^ Hence, half of infants with symptomatic inherited arrhythmias syndromes would be expected to present *in utero*, often with 2:1 block. Using foetal magnetocardiography in 39 foetuses, LQTS Type 1 and type 2 were rarely associated with AV block (6%).^11^ However, among those with LQTS type 3 and those with rare variants, AV block was seen in 7/12 cases and was associated with torsades de pointes (TdP) in 3/7, all with *de novo* mutations. Chivers *et al*. in a recent metanalysis of 83 studies and 265 foetuses presenting *in utero* with LQTS, found that 2:1 AV block, TdP, and a lack of a family history of LQTS (*de novo* genetic status) predicted a high risk of mortality.^12^ Thus, if second degree AV block is seen *in utero*, it is important to try to identify the aetiology, and to risk stratify the patient using isovolumetric relaxation time,^13^ foetal magnetocardiography (if available), and genetic testing. If the clinical situation proves consistent with LQTS, one can implement *in utero* clinical risk-reduction strategies, such as correction of low maternal magnesium, calcium, or vitamin D, and limiting exposure to QTc-prolonging medications.^14^ TdP has been successfully treated *in utero* with beta blockers and lidocaine. Yet, it is reassuring to know that despite early presentation, infants with second degree AV block do well long-term. However, since ascertainment assumed a live birth, lethal *de novo* foetal cases may not be represented in the Miycinski *et al*. study. The role of early pacing should be further investigated through review of the Chivers *et al*. data^12^ and these investigators to further assess its impact, if any, in producing long-term survival in those with LQTS.

In 2015, the principal author, Baruteau *et al.*, wrote an excellent review on progressive conduction system disorders that present during childhood. He outlined nine genes that were involved in ion channel function, gap junction communication, and cardiac transcription.^15^ To his list of nine disorders, we would add HCN4, reported in 2020.^16^

In the present paper, the authors call for greater delineation of genetic conduction system disorders affecting the foetus, infants, and children. We would echo that call. With continued effort to obtain genetic assessment in unusual phenotypes, such as unexplained stillbirth, SIDS, and sudden unexplained death syndrome, we will reduce the number of genetically negative inherited arrhythmias syndrome and AV block and expand our knowledge of familial progressive conduction system disease. Such phenotype–genotype correlations in early life would do much to overcome genetic registries biased to the adult survivor, and to more precisely classify rare variants that are ‘one-off’ cases and shrink the numbers of variants of uncertain significance. We learned recently that the lifetime risk for those with genetically negative LQTS is not different from those that are genetically positive,^17^ suggesting that many patients have the potential to be under-recognized and consequently, under-treated. The authors conclude appropriately that genetic testing using arrhythmia and cardiomyopathy gene panels should be considered in every infant or child with cardiac conduction system disease whose mothers are anti-Ro/SSA negative. This should be followed with cascade family screening when positive. We commend the authors on collaboration and their career-long efforts to clarify the causes and prognosis of conduction disease in childhood.
